# Lack of IgA envelope-reactive antibody producing cells in terminal ileum in early and chronic HIV-1 infection

**DOI:** 10.1186/1742-4690-9-S2-P201

**Published:** 2012-09-13

**Authors:** AM Trama, H Liao, A Foulger, DJ Marshall, JF Whitesides, R Parks, R Meyerhoff, KE Lloyd, M Donathan, J Lucas, K Soderberg, TB Kepler, N Vandergrift, N Yates, GD Tomaras, MA Moody, BF Haynes

**Affiliations:** 1 Duke University, Durham, NC, USA; 2 Boston University, Boston, MA, USA

## Background

HIV-1 vaccines must induce protective antibodies at mucosal surfaces; the role of IgA in protection remains unknown. The HIV-1 Env antibody response begins ~day 17 after transmission, and derives from a polyreactive memory B cell pool of gut flora-reactive IgG1 and IgA B cells. Whereas the IgG Env antibody response persists years after acute HIV-1 infection, the initial IgA response decreases over the first month. There is also selective destruction of terminal ileum germinal centers in early HIV-1 infection (EHI). To determine HIV-1 IgA responses in gut, we isolated Env-reactive antibodies from ileum from patients in EHI and chronic HIV-1 infection (CHI).

## Methods

Single plasma cells (PCs) and IgD- memory B cells were sorted from the ileum and/or blood of 7 EHI and 3 CHI. Antibodies were isolated by PCR amplification of Ig heavy chain V(D)J and light chain VJ genes and characterized by ELISA and Luminex.

## Results

Whereas CHI blood memory IgA+ B cells reactive with HIV-1 envelope ranged from 0.20-0.79%, only 0-0.07% of ileum IgA+ B cells were Env-reactive. Of 254 mAbs isolated from EHI ileum, only 3 (1.2%) were HIV-1-reactive. In CHI, 9 (5.7%) of 158 mAb were HIV-1 reactive. None of the HIV-1 reactive ileum antibodies were of the IgA isotype.

## Conclusion

HIV-1 envelope reactive IgA+ memory B cells and PCs can be found in the blood, but there is a dearth of HIV-1 reactive memory IgA+ B cells and PCs in ileum in EHI and CHI. Loss of IgA in plasma after acute HIV-1 infection is paralleled by the loss of IgA+ B cells in ileum, and is likely a consequence of HIV-1-induced ileum germinal center apoptosis. For vaccine design, it will be important to determine if mucosal IgA+ B cell loss is due to replicating virus or is triggered by soluble HIV-1 envelope.

